# Badminton expertise shapes cue extraction and evidence accumulation during perceptual prediction

**DOI:** 10.3389/fpsyg.2026.1828824

**Published:** 2026-06-03

**Authors:** Qiong Wu, Bo Zhao, Zhenqiang Zhang, Xin Liu, Zhihui Liu, Lin Wu, Wenzi Wang

**Affiliations:** 1 Hebei Energy College of Vocation and Technology, Tangshan, China; 2 Tangshan Research Institute of Beijing Jiaotong University, Tangshan, China; 3 School of Psychology, Shanghai University of Sport, Shanghai, China

**Keywords:** badminton, drift diffusion model, evidence accumulation, perceptual prediction, temporal occlusion

## Abstract

**Objective:**

This study aims to investigate the impact of badminton-specific experience on the mechanisms of cue extraction and processing during perceptual prediction from a computational modeling perspective.

**Methods:**

Thirty-six participants (18 experts and 18 novices) were recruited for the experiment. Utilizing a temporal occlusion paradigm, three cue stages were established: 40 milliseconds before racket-shuttlecock contact (T1), at contact (T2), and 40 milliseconds after contact (T3). Participants were tasked with predicting the landing location of the shuttlecock. The study not only recorded behavioral metrics (accuracy and reaction time) but also employed the Drift Diffusion Model (DDM) to jointly model the behavioral data, extracting three latent processing parameters: drift rate, decision threshold, and non-decision time.

**Results:**

Accuracy analysis revealed significant main effects for cue stage and group, alongside a significant interaction. Simple main effect analysis between groups indicated that experts’ accuracy at T1 and T2 was significantly higher than that of novices, whereas the difference was non-significant at T3. Reaction time analysis also showed significant main effects for cue stage and group, alongside a significant interaction; experts exhibited significantly shorter reaction times than novices across all three time points. DDM results demonstrated that experts had a significantly higher drift rate than novices at T1 and T2, while this inter-group difference disappeared at T3. As information increased, experts showed a more pronounced upregulation in the decision threshold at T3, whereas novices exhibited greater threshold fluctuations. Non-decision time was consistently shorter for experts across all stages.

**Conclusion:**

The optimization of perceptual prediction mechanisms driven by badminton-specific experience exhibits stage specificity. The expert advantage is primarily concentrated in the early processing of kinematic cues when information is incomplete, manifesting as a higher rate of evidence accumulation. In stages with more abundant information, experts maintain output stability through an optimized dynamic speed-accuracy tradeoff (threshold upregulation) and retain shorter non-decision times across all stages. This suggests that long-term training facilitates the automated synergy of stimulus encoding and motor preparation.

## 1 Introduction

Badminton is a prototypical high-speed interception sport: shuttle velocity is high and trajectories and landing locations vary rapidly across rallies. Players therefore often must predict the incoming path and landing point—and initiate an appropriate response—under incomplete information, such as pre-contact body posture, racquet orientation at contact, and early post-contact flight. Recent work on perceptual–cognitive expertise suggests that elite performance is not simply a matter of “seeing faster,” but reflects more efficient extraction of diagnostic cues, better use of contextual probabilities and more effective evidence integration under severe time pressure (DeCouto et al., 2024).

Anticipation is both a key component of sport-specific performance and a robust marker of perceptual–cognitive advantages across skill levels. The temporal-occlusion paradigm is widely used to characterize anticipation by cutting off video information at critical moments (for example, before contact, at contact and after contact) to manipulate available cues and quantify prediction efficiency and accuracy (Song et al., 2025). In badminton, video-based temporal-occlusion studies similarly report reliable expertise-related differences in prediction accuracy and speed, with effects that vary across occlusion stage and cue type (Robertson-Martens et al., 2022; De Waelle et al., 2023).

Recent systematic reviews and meta-analyses report overall moderate-to-large expert-novice differences in anticipation and positive effects of temporal-occlusion-based training, but they also highlight heterogeneity across sports, task types, training protocols and transfer tests (Müller et al., 2024; Song et al., 2025). These findings imply that expert advantages may arise from both more effective cue pick-up and more efficient cue processing/integration, in a manner that changes dynamically with the information available at each stage, while the size and transferability of these effects should be interpreted in relation to task representativeness. Moreover, an updated synthesis of mobile eye tracking and natural gaze behavior highlights that, in more representative sport contexts, gaze strategies and information sampling are highly dynamic; overall accuracy and mean reaction time alone often fail to reveal the underlying processing architecture (Kredel et al., 2023).

A complementary action-observation and motor-expertise literature further suggests that sport-specific prediction depends not only on visual experience but also on the observer’s motor repertoire and internal motor representations. Elite athletes can predict action outcomes earlier and more accurately than novices or expert observers with comparable visual familiarity, and this advantage has been linked to motor-resonance and action-observation mechanisms during sport-specific tasks (Aglioti et al., 2008; Smith, 2016). Foundational accounts of the mirror-neuron system propose a neural substrate for action understanding and motor simulation (Rizzolatti and Craighero, 2004), and recent rugby and soccer studies indicate that prediction accuracy is finely tuned by position-specific motor expertise and training history (Paolini et al., 2023; Paolini et al., 2025). This framework is directly relevant to badminton anticipation, because pre-contact body and racquet kinematics must be mapped rapidly onto likely action outcomes.

From an information perspective, anticipation does not rely solely on kinematic cues (for example, the spatiotemporal dynamics of an opponent’s trunk, upper limb and racquet), but is also shaped by non-kinematic contextual priors (such as opponent tendencies, tactical context and probabilistic information). Notably, explicit contextual information does not always improve performance under uncertainty: when information is unreliable or inconsistent with the unfolding action, it can even impair performance—sometimes more strongly in highly skilled athletes—indicating that cue weighting depends on both uncertainty and expertise (Magnaguagno et al., 2022). If anticipation is framed as inference about future states under incomplete information, predictive processing and active inference provide a unifying computational perspective: athletes must weight priors and sensory evidence by their reliability to form optimal predictions (Harris et al., 2022; Gredin et al., 2023).

Within this framework, anticipation approximates Bayesian inference in uncertain environments. Individuals use an internal generative model to integrate contextual priors (for example, strategic tendencies and probability cues) with current sensory evidence (for example, kinematic cues and early flight information), weighting each source by reliability to minimize prediction error and guide action preparation (Harris et al., 2022). Empirical sport studies support this prior–evidence integration account: under high uncertainty, explicit contextual information tends to benefit performance only when it is sufficiently reliable and congruent with the impending action; misleading or incongruent information can disrupt behavioral control more strongly in experts, suggesting that experts are not simply “more prior-driven” but are more sensitive to reliability and congruency (Magnaguagno et al., 2022). Neurocognitive evidence further indicates separable expertise-related differences in processing time courses and dependence strategies when integrating priors and kinematic cues (Wang et al., 2025). Consistent with this, experts can preserve strategic use of priors under time constraints by adapting processing strategies to task demands (Lu et al., 2025). We do not assume a one-to-one mapping between Bayesian precision weighting and DDM parameters. Instead, we use the DDM as a complementary process model that decomposes behavior into evidence accumulation, decision caution and non-decision components, which can serve as behavioral markers that are consistent with, but not direct measures of, more efficient internal motor representations and action-observation mechanisms.

Within the badminton-specific anticipation literature, a key methodological limitation is the reliance on accuracy and reaction time as separate outcome measures. Although these metrics capture performance differences, they cannot determine whether effects are driven by evidence accumulation efficiency, speed–accuracy trade-off policies, or non-decision processes such as perceptual encoding and motor execution. Evidence accumulation models provide a more mechanistic approach. In particular, the drift–diffusion model (DDM) jointly accounts for accuracy and reaction time, separating latent parameters that reflect evidence quality/accumulation efficiency (drift rate), decision caution and speed–accuracy trade-offs (boundary separation), and non-decision time encompassing encoding and motor processes (Myers et al., 2022; Henrich et al., 2024). Importantly, computational work in other sports has already used hierarchical diffusion or Bayesian models to explain split-second decisions and the integration of contextual and kinematic information under extreme time pressure, including handball penalty and deception paradigms (He et al., 2025; Weinberg et al., 2025). To our knowledge, however, no study has yet applied hierarchical drift-diffusion modeling to badminton anticipation across temporal-occlusion stages. The present study therefore extends process-level modeling to a new sport-specific context rather than claiming that anticipation research in general lacks computational decomposition.

Here, we adopt a computational modeling perspective in a badminton video-based anticipation task that manipulates available cue information and contrasts experts with novices. We apply the DDM to behavioral data to characterize how drift rate, boundary separation and non-decision time vary across cue stages. Specifically, we ask: (i) does expert advantage primarily reflect higher evidence accumulation efficiency (drift rate) or more adaptive speed–accuracy trade-offs (boundary modulation)? (ii) as cues accumulate from pre-contact to post-contact, do expert–novice differences converge or diverge across stages? and (iii) is badminton expertise associated with shorter non-decision processes, consistent with more automated perceptual encoding and response execution? By linking anticipation performance to computationally defined parameters, this work aims to provide testable markers for mechanistic interpretation and training design.

## 2 Materials and methods

### 2.1 Participants

Thirty-six participants were assigned to an expert group or a novice group based on badminton experience (18 per group). The expert group (10 men, 8 women) had a mean age of 22 ± 1.16 years and a training history of 6.68 ± 1.85 years; 10 were national first-class athletes and 8 were national second-class athletes. Experts met the following criteria: (i) ≥5 years of formal training and (ii) sustained training over the previous 3 years at ~4 sessions per week, ~3 h per session. The novice group (11 men, 7 women; 21 ± 1.15 years) had no formal training experience, no professional training in other interception sports (for example, table tennis or tennis), and only occasional or no viewing experience of interception-sport competitions. All participants were healthy, reported no psychiatric disorders, had normal or corrected-to-normal vision and no color-vision deficiencies, and participated voluntarily. The study was approved by the Ethics Committee of Shanghai University of Sport (No. 102772025RT300), and all participants provided written informed consent.

### 2.2 Design

We used a 2 (experience: expert, novice) × 3 (cue stage: T1, T2, and T3) mixed design. Experience was a between-subject factor and cue stage was a within-subject factor. T1 ended 40 ms before racquet–shuttle contact (one frame before contact), T2 ended at contact and T3 ended 40 ms after contact (one frame after contact). Dependent variables were accuracy, reaction time and DDM parameters.

### 2.3 Stimulus materials

To approximate match-like perceptual conditions and provide a realistic anticipation viewpoint, stimuli consisted of custom-recorded videos. A shuttle-launching machine delivered high clear shots on a badminton court, and a receiver on the opposite side returned the shuttle with a forehand drop shot. The receiver was a 23-year-old male national first-class athlete (right-handed). A single hitter was used to reduce between-model kinematic variability and keep the occlusion manipulation consistent across trials. This choice increases internal control but limits ecological generalisability, especially because male and female badminton players can differ in movement kinematics and neuromuscular control (Hu et al., 2023); consequently, the present design was not intended to test sex-related observer-by-stimulus effects. Two shot outcomes were recorded: straight drop shots and cross-court drop shots. Videos were recorded using a Sony a7M3 camera (1920 × 1,080 pixels; 120 frames per second). The camera was placed on the server side and positioned to approximate the perspective of the opposing player. Eighty video sequences were recorded.

Videos were edited in Adobe Premiere. Based on overall clarity and the visibility of the key frame capturing racquet–shuttle contact, videos were exported at 1280 × 720 pixels and converted to 25 frames per second, yielding a frame duration of 40 ms. The hitter’s face was pixelated to protect identity. Task difficulty was adjusted in a pilot study to ensure that accuracy could discriminate experts from novices; after screening, 20 video sequences were retained. The retained clips were balanced across the two outcomes and landing sides (10 straight/cross-court and 10 left/right trials in the base set). Following prior work, we generated stimuli at three occlusion points: T1 (40 ms before contact; one frame before contact), T2 (at contact) and T3 (40 ms after contact; one frame after contact). For each participant, every base clip appeared three times at each occlusion stage, yielding 20 clips × 3 repetitions = 60 trials per stage and 180 trials in total. This repeated-clip structure was necessary for stable DDM estimation, but it may also have increased stimulus familiarity across the task and is therefore considered in the Discussion and limitations.

### 2.4 Procedure

**Figure 1:**
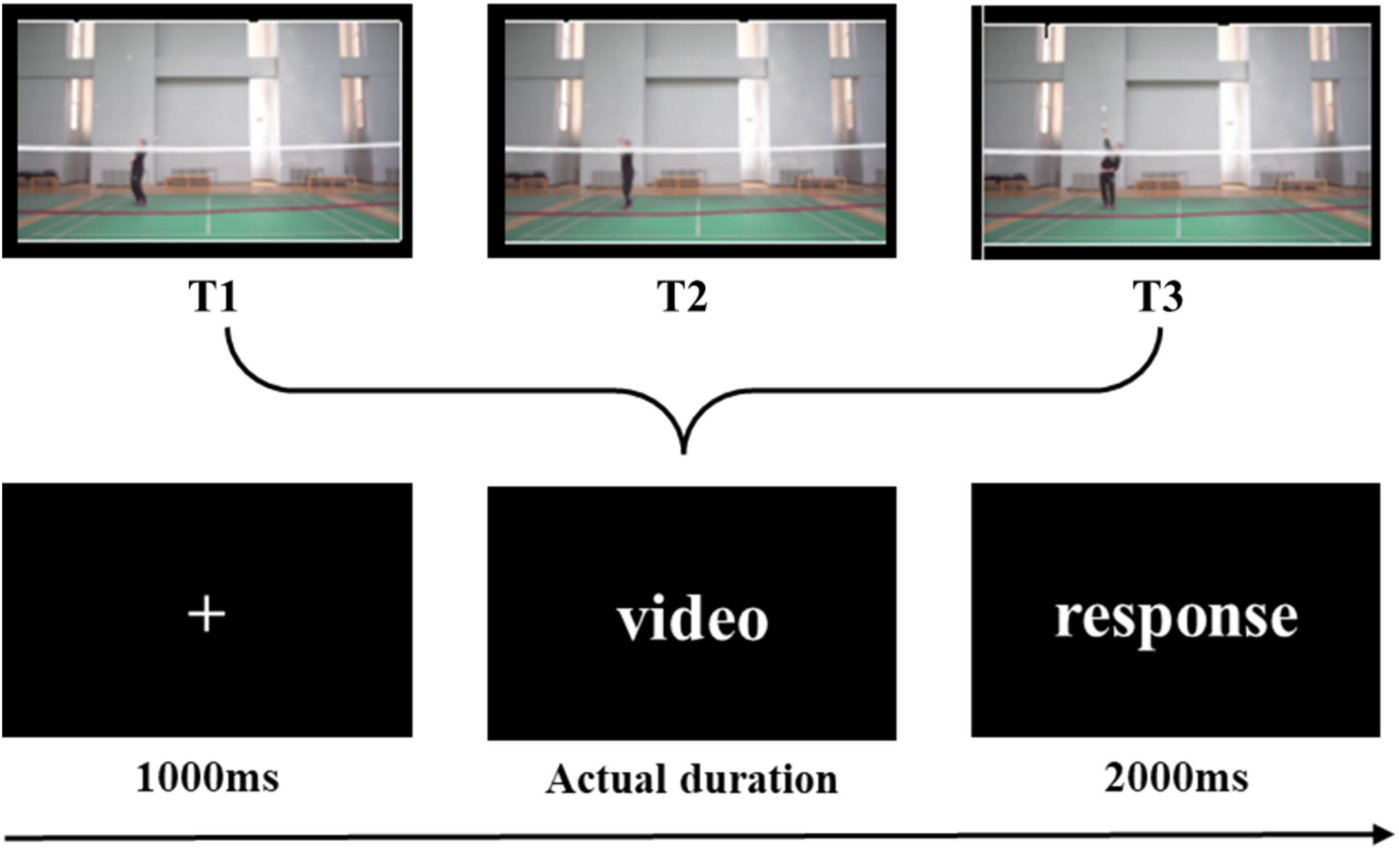
Experimental trial procedure. T1 ended 40 ms before racquet–shuttle contact (one frame before contact), T2 ended at contact, and T3 ended 40 ms after contact (one frame after contact).

Testing was conducted in a quiet, sound-attenuated and light-controlled room. Participants completed the task in a comfortable seated position. Before the experiment, the experimenter explained the procedure and participants completed a demographic questionnaire. Stimuli were presented on a 13-inch laptop display. The experiment comprised three blocks of 60 trials each (180 trials total). Trials were pseudorandomised in stratified lists so that cue stage and landing side were balanced within each block and no more than three consecutive trials shared the same cue stage or landing side. Block order was not tied to cue stage, and straight/cross-court outcomes were randomized within the same stratified lists. Each trial began with a 1,000-ms fixation, followed by the occluded video. A response screen then appeared for 2,000 ms, during which participants indicated the predicted landing side (left vs. right) using the keyboard: F for left and J for right (Figure 1). Reaction time was measured from the onset of the response screen to the first valid keypress. Participants rested for 1–2 min between blocks. The session lasted ~15–20 min. The task was implemented in E-Prime 2.0, which recorded reaction time and accuracy.

### 2.5 Data acquisition and analysis

#### 2.5.1 Behavioral data

Valid data were obtained from all 36 participants (18 experts and 18 novices). Accuracy and reaction time were recorded in E-Prime 2.0 and exported to Excel for preprocessing. Incorrect responses were retained because accuracy was a dependent variable. Trials were classified as anomalous and excluded only when no response was made within the 2,000-ms response window, more than one key was pressed, or RT was physiologically implausible (<200 ms). Furthermore, we excluded the trial sessions whose reaction times were beyond ±3 standard deviations. We then conducted 2 × 3 mixed repeated-measures ANOVAs (cue stage as the within-subject factor; group as the between-subject factor) separately for accuracy and reaction time in SPSS 21.0. *Post hoc* comparisons used Bonferroni correction. Statistical significance was set at *p* < 0.05.

#### 2.5.2 Drift–diffusion modeling

We fitted hierarchical Bayesian DDMs using dockerHDDM v1.0.1 in Python 3.9 (Pan et al., 2025). Because left and right landing locations were equiprobable, the task was symmetric; we therefore fixed the starting point at 0.5 rather than estimating it as a free parameter (Gold and Shadlen, 2007; Hanks et al., 2006). This constraint reduced model complexity and matched the balanced design, but it also means that possible response biases related to expertise or occlusion stage were not estimated directly. We focused on three parameters: drift rate (v), boundary separation (*α*) and non-decision time (*τ*). Competing models differed in which parameters were allowed to vary across conditions. Posterior distributions were estimated using hierarchical Bayesian inference with Markov chain Monte Carlo sampling. For each model, we ran four chains with 10,000 samples per chain and discarded the first 2,000 samples as burn-in. Convergence was assessed using the Gelman–Rubin statistic (R-hat), with values < 1.1 taken to indicate acceptable convergence. Model selection used the deviance information criterion (DIC), with lower values indicating better fit (Wiecki et al., 2013). To test condition effects, we compared group-level posterior distributions using 95% highest density intervals (HDIs); an HDI that excluded zero was interpreted as evidence for a credible difference (Johnson et al., 2017). For interpretability, posterior means and 95% highest-density intervals (HDIs) were computed from the posterior samples for drift rate (v), boundary separation (*α*), and non-decision time (*τ*) for each group × cue-stage combination.

## 3 Results

### 3.1 Behavioral performance

#### 3.1.1 Accuracy

Descriptive statistics are presented in Table 1. A 2 (group) × 3 (cue stage) mixed ANOVA showed a significant main effect of cue stage [*F*(2,68) = 148.572, *p* < 0.001, *η*_p_^2^ = 0.814], a significant main effect of group [*F*(1,34) = 33.116, *p* < 0.001, *η*_p_^2^ = 0.493], and a significant interaction [*F*(2,68) = 17.988, *p* < 0.001, *η*_p_^2^ = 0.346]. Within the novice group, accuracy did not differ between T1 and T2, but differed between T2 and T3 (*p* < 0.001). Within the expert group, accuracy differed pairwise across all stages (all *p* < 0.05). Between groups, experts were more accurate than novices at T1 (*p* < 0.001) and T2 (*p* < 0.001), whereas no group difference was observed at T3.

**Table 1:** Accuracy of different groups under different cue conditions (%).

Group	Cue	Average value ± Standard error
Novice group	T1	58.8111 ± 10.44987
	T2	61.1389 ± 9.92241
	T3	93.2333 ± 5.16299
Expert group	T1	72.2556 ± 8.26276
	T2	80.8389 ± 6.91419
	T3	93.0389 ± 7.31012

#### 3.1.2 Reaction time

Descriptive statistics are presented in Table 2. Reaction time showed a significant main effect of cue stage [*F*(2,68) = 6.451, *p* < 0.05, *η*_p_^2^ = 0.159], a significant main effect of group [*F*(1,34) = 14.851, *p* < 0.001, *η*_p_^2^ = 0.304], and a significant interaction [*F*(2,68) = 3.448, *p* < 0.05, *η*_p_^2^ = 0.092]. Within the novice group, reaction time did not differ between T1 and T2, but differed between T2 and T3 (*p* < 0.001). Within the expert group, reaction time did not differ across the three stages. Between groups, experts responded faster than novices at all three stages (all *p* < 0.05). Because the group × cue-stage interaction was small, this effect should be interpreted cautiously and mainly reflects the T2-to-T3 speeding observed in novices.

**Table 2:** Reaction time of different groups under different cue conditions (ms).

Group	Cue	Average value ± Standard error
Novice group	T1	1434.372 ± 62.947
	T2	1367.933 ± 60.567
	T3	1258.250 ± 53.152
Expert group	T1	1073.928 ± 62.947
	T2	1055.144 ± 60.567
	T3	1045.222 ± 53.152

### 3.2 Drift–diffusion model results

We fitted hierarchical DDMs with group (expert vs. novice) as a between-subject factor and cue stage (T1/T2/T3) as a within-subject factor. Competing models were compared in terms of free parameters, convergence and goodness of fit (DIC; Table 3). Model 6 yielded the lowest DIC and acceptable convergence and was selected as the best-fitting model for inference.

**Table 3:** Model convergence and fit.

Model	Free parameters	Novice (max R-hat)	Novice (DIC)	Expert (max R-hat)	Expert (DIC)
1	α	1.443	3433.82	1.002	1554.17
2	α, τ	1.004	3380.36	1.003	1556.73
3	τ	1.002	3520.23	1.003	1554.23
4	v	1.278	2845.96	1.001	1415.40
5	v, α	1.070	2835.81	1.003	1442.60
6	v, α, τ	1.004	2722.35	1.005	1373.40
7	v, τ	1.004	2778.28	1.005	1402.73
8	none	1.001	3560.45	1.001	1553.09

Overall, drift rate increased with increasing information for experts (Table 4). For novices, drift rate did not differ between T1 and T2, but was higher at T3 than at both T1 and T2. For boundary separation, experts showed higher boundaries at T3 than at T1 and T2, whereas T1 and T2 did not differ. Novices showed a lower boundary at T2 than at T1, and a higher boundary at T3 than at T2, indicating greater stage-to-stage fluctuation. For non-decision time, novices showed no reliable stage differences. In experts, non-decision time was shorter at T3 than at T1, while the T2 − T1 and T3 − T2 contrasts did not exclude zero.

**Table 4:** Posterior contrasts across conditions (mean and 95% HDI).

Group/contrast	Drift rate (M)	Drift rate (95% HDI)	Boundary (M)	Boundary (95% HDI)	Non-decision (M)	Non-decision (95% HDI)
Novice T2 − T1	0.09	[−0.10, 0.29]	−0.16*	[−0.24, −0.08]	0.04	[−0.05, 0.13]
Novice T3 − T1	3.20*	[2.27, 4.26]	0.33	[−0.12, 1.00]	0.03	[−0.08, 0.15]
Novice T3 − T2	3.10*	[2.14, 4.14]	0.49*	[0.02, 1.15]	−0.01	[−0.16, 0.13]
Expert T2 − T1	0.48*	[0.23, 0.76]	0.06	[−0.04, 0.15]	−0.007	[−0.09, 0.07]
Expert T3 − T1	3.04*	[1.85, 4.31]	2.16*	[0.76, 3.61]	−0.13*	[−0.22, −0.03]
Expert T3 − T2	2.55*	[1.28, 3.78]	2.10*	[0.72, 3.58]	−0.12	[−0.24, 0.006]
T1 Expert − Novice	0.61*	[0.36, 0.87]	−0.20*	[−0.35, −0.04]	−0.20*	[−0.41, −0.002]
T2 Expert − Novice	1.00*	[0.65, 1.37]	0.02	[−0.15, 0.20]	−0.25*	[−0.49, −0.02]
T3 Expert − Novice	0.45	[−1.16, 2.05]	1.63*	[0.15, 3.31]	−0.36*	[−0.62, −0.12]

T1/T2/T3 correspond to increasing information (low → high). * indicates the reported 95% HDI excludes 0.

Between-group comparisons showed that experts had higher drift rates at T1 and T2, whereas the group difference disappeared at T3. Boundary separation was lower for experts at T1, showed no group difference at T2, and was higher for experts at T3, consistent with a larger boundary increase in experts as information increased. Non-decision time was shorter in experts than in novices at all stages.

To complement the contrast-based summaries, we examined the absolute posterior estimates of the diffusion-model parameters for each group × cue-information stage (Table 5). Across both groups, drift rate (v) increased as cue information became richer, with the highest within-group posterior estimates observed at T3. In novices, v increased from 0.327 at T1 to 3.525 at T3; in experts, v increased from 0.941 at T1 to 3.978 at T3. Boundary separation (*α*) showed a more moderate stage-dependent pattern in novices, increasing from 1.352 at T1 to 1.686 at T3, whereas experts showed a stronger increase from 1.157 at T1 to 3.313 at T3. Non-decision time (*τ*) was consistently shorter in experts than in novices across cue stages. Together, these absolute posterior summaries complement the posterior contrasts reported in Table 4 and make the overall magnitude and pattern of the model parameters more transparent.

**Table 5:** Absolute posterior estimates of DDM parameters by group and cue-information stage.

Group	Cue stage	*v*	*α*	*τ*
Novice	T1	0.327 [0.179, 0.483]	1.352 [1.268, 1.439]	0.972 [0.827, 1.116]
Novice	T2	0.421 [0.222, 0.640]	1.197 [1.096, 1.298]	1.011 [0.846, 1.179]
Novice	T3	3.525 [2.572, 4.571]	1.686 [1.205, 2.332]	1.000 [0.818, 1.186]
Expert	T1	0.941 [0.745, 1.145]	1.157 [1.036, 1.284]	0.768 [0.625, 0.913]
Expert	T2	1.424 [1.137, 1.711]	1.215 [1.071, 1.358]	0.761 [0.604, 0.925]
Expert	T3	3.978 [2.754, 5.232]	3.313 [1.942, 4.810]	0.641 [0.467, 0.811]

Values are posterior mean [95% HDI]. A1 denotes the novice group and A2 denotes the expert group. T1, T2 and T3 denote the least, moderate and most informative cue stages, respectively. v = drift rate; α = boundary separation; τ = non-decision time.

## 4 Discussion

Using a temporal-occlusion paradigm that controlled cue availability (pre-contact, contact and post-contact), we examined how badminton-specific experience shapes cue extraction and processing during perceptual prediction from a computational modeling perspective. Behaviorally, experts outperformed novices at T1 (40 ms before contact) and T2 (at contact), whereas the accuracy advantage disappeared at T3 (40 ms after contact). Computationally, experts showed higher drift rates and shorter non-decision times at T1/T2, with drift rates converging at T3; experts also showed a larger boundary increase at T3, whereas novices exhibited more variable boundaries. Together, these findings suggest that expertise-related differences are stage-specific and are most visible when information is incomplete. Importantly, the DDM parameters provide a process-level behavioral decomposition rather than direct evidence for a single cognitive, motor or neural mechanism.

From the perspective of information structure and uncertainty, T1 and T2 are dominated by kinematic cues with limited diagnosticity, such that judgments likely rely more strongly on internal action–outcome mappings and rapid reliability assessment. At T3, early flight information becomes available and is substantially more diagnostic; novices can therefore compensate using more direct outcome-related cues, which may contribute to the convergence in accuracy. This interpretation is consistent with Bayesian accounts in which expert–novice differences are most evident under higher uncertainty, when internal models guide inference, and are compressed when external evidence becomes sufficiently reliable (Harris et al., 2023). However, the high T3 accuracy in both groups (approximately 93%) and repeated exposure to the same 20 clips may also have produced ceiling and familiarity effects. Therefore, the T3 convergence should be interpreted as reflecting richer sensory evidence together with possible task-ease and learning/familiarity contributions, rather than as a pure demonstration that external evidence overrides expertise-related internal models.

The concentration of expert advantages at T1/T2 is consistent with the heavy reliance of racket sports on pre-contact and contact kinematics (trunk, hitting arm and racquet). For example, in tennis occlusion tasks that manipulate visibility of key body regions, occluding the trunk or ball can markedly reduce anticipation, alongside changes in neural dynamics related to body processing and perception–action integration; higher-skilled athletes show stronger engagement at stages linking sensory encoding to response execution (Costa et al., 2023). This aligns with the present drift-rate advantage at T1/T2, suggesting more efficient transformation of limited kinematic input into discriminative evidence.

Prior eye-tracking work provides a plausible behavioral route for this early-stage advantage. In badminton, higher-level players show faster responses and higher judgment quality, together with more efficient allocation of visual attention and more stable information processing during attentional shifts, whereas lower-level players are more susceptible to external interference (Chen et al., 2023). Mapped onto the present modeling results, a testable hypothesis is that experts’ higher drift rates at T1/T2 partly reflect more efficient sampling of diagnostic regions of interest and higher-quality cue pick-up per unit time.

The present pattern also fits naturally with action-observation and mirror-mechanism accounts of sport expertise. In basketball, elite athletes anticipate shot outcomes earlier and more accurately than novices and expert watchers, and this behavioral advantage is accompanied by temporally specific motor resonance during action observation (Aglioti et al., 2008). A systematic review similarly concluded that expert athletes generally outperform novices during sport-specific action anticipation tasks and that fMRI, EEG and TMS studies often implicate action-observation and motor networks, although the precise neural loci vary across studies (Smith, 2016). From this perspective, the higher early drift rates and shorter non-decision times observed here can be interpreted as computational markers consistent with more efficient recruitment of internal motor representations during the observation of badminton-specific kinematics.

Drift rate showed a clear stage dependency. Because drift rate captures the combined quality of evidence and the efficiency of extracting and integrating that evidence, higher drift rates in experts at T1/T2 are consistent with the idea that experts convert limited kinematic cues into stronger directional evidence within a shorter window. In contrast, novices showed a pronounced increase only at T3, suggesting that their accumulation process depends more strongly on post-contact flight cues. Similar modeling work in sport anticipation has demonstrated that hierarchical diffusion models can disentangle systematic changes in accumulation and strategic parameters across information conditions, clarifying which components contribute to expertise effects (He et al., 2025). However, drift rate does not uniquely isolate cue extraction from other contributors such as strategic base-rate expectations, familiarity with repeated video clips, or general experience with video-based tasks. Thus, we interpret the drift-rate pattern as model-based evidence consistent with efficient cue extraction and action-outcome mapping, not as a direct measure of visual cue pick-up.

Boundary separation changes highlight model-based differences in speed–accuracy trade-off policies. Experts showed a marked boundary up-regulation at high information (T3), whereas novices’ boundaries fluctuated more across stages. This pattern is consistent with the interpretation that experts adjusted decision caution as cue reliability changed, favoring greater caution when evidence was richer to stabilize output. However, T3 was also the easiest condition and approached ceiling accuracy, and repeated clips may have increased familiarity; these factors could also influence boundary estimates. In addition, the present task is a laboratory-based video occlusion task with button-press responses and therefore occupies a low level of perception–action coupling in the hierarchy proposed by Huesmann and Loffing (2024). The current boundary results therefore provide a useful starting point, but more representative tasks involving step initiation, directional movement selection or interactive VR hitting are needed to determine whether similar boundary modulation occurs under on-court control demands.

Group differences in non-decision time extend the model-based account to processes outside the accumulation stage. Experts showed shorter non-decision times at all stages, which is consistent with faster stimulus encoding and/or response implementation. Integrated decision–action diffusion theories propose that accumulation can continuously drive motor preparation, such that movement preparation begins before the decision terminates via gated release mechanisms (Servant et al., 2021; Dendauw et al., 2024). Nevertheless, because the present task used simple keypress responses and did not include EMG, movement kinematics or neural measures, non-decision time cannot distinguish perceptual encoding from motor execution. The observed non-decision advantage should therefore be viewed as a computational marker consistent with more automated stimulus–response processing, not as direct evidence for gated motor release.

These results suggest two training implications. First, training priorities should shift earlier, targeting the use of pre-contact and contact kinematic cues to improve the evidence-accumulation efficiency reflected in drift rate. Second, training should manipulate information richness while controlling task constraints and load to foster more stable boundary adjustment and more efficient perception–action coupling. Systematic reviews indicate that video-based perceptual–cognitive training can improve anticipation and decision performance, and that first-person perspectives and more immersive delivery (including VR) may increase representativeness and transfer (Zhao et al., 2022). In badminton, depth cues influence spatial inhibition and attention, and badminton players appear more sensitive to depth information (Liu et al., 2024), suggesting that incorporating three-dimensional depth and dynamic cues may better approximate match information structure. In addition, multisensory guidance (for example, sonification) can alter gaze patterns and facilitate anticipation learning in novices (Khalaji et al., 2022), offering a practical route to combine attentional guidance with occlusion-based training.

Several limitations motivate future work. First, the current stimuli were restricted to drop shots produced by a single male right-handed hitter, and participants made a binary left–right judgment. This controlled design improved internal validity but constrains generalisability across players, sexes, handedness, stroke types and deceptive actions. Future studies could include multiple male and female models, broader stroke repertoires and deception manipulations, and use hierarchical diffusion models to test interactions among deception, cue stage and experience (He et al., 2025). Second, our interpretation of cue extraction is currently parameter-based; future designs should jointly measure gaze (regions of interest), kinematics, EMG and action-initiation indices to establish reproducible mappings from input cues to accumulation parameters (Chen et al., 2023). Third, the task used a flat-screen video-occlusion paradigm with keypress responses and therefore has low perception–action coupling. More representative movement-based or VR paradigms are needed to examine whether drift rate, boundary separation and non-decision time show comparable patterns when anticipation is coupled to on-court movement. Fourth, high T3 accuracy and repeated exposure to 20 clips may have produced ceiling and familiarity effects; future analyses should model trial order or compare early versus late trials to quantify learning across the experiment. Fifth, the starting point was fixed at 0.5 and the selected DDM was not cross-validated in an independent sample; future work should test sensitivity to starting-point assumptions and use bootstrapping, posterior predictive checks or independent replication to strengthen robustness. Finally, recent EEG-HDDM work provides a direct pathway to extend the present behavioral-computational approach toward neuro–computational coupling and to test how semantic, kinematic and contextual priors jointly support rapid prediction (Mou et al., 2025; Yan et al., 2025).

## 5 Conclusion

Badminton-specific experience was associated with stage-specific differences in perceptual prediction. The expert advantage was concentrated at T1 and T2, when information was incomplete and kinematic cues were relatively uncertain, whereas accuracy and drift rate converged at T3 when post-contact information was available. This convergence likely reflects richer external evidence together with possible ceiling and stimulus-familiarity effects. Computational modeling suggests that the early expert advantage is consistent with a higher evidence accumulation rate and shorter non-decision time, and that the T3 pattern is consistent with greater decision caution in experts. These DDM parameters should be interpreted as behavioral-computational markers of evidence accumulation, decision policy and non-decision processing rather than as direct demonstrations of specific neural or motor mechanisms. Future work should extend this low-coupling video paradigm to movement-based and VR tasks, include multiple hitter models and stroke types, manipulate contextual priors explicitly, and combine DDM with eye-tracking, kinematic, EMG and neurophysiological measures.

## Data Availability

The original contributions presented in the study are included in the article/supplementary material, further inquiries can be directed to the corresponding author.

## References

[ref1] Aglioti S. M. Cesari P. Romani M. Urgesi C. (2008). Action anticipation and motor resonance in elite basketball players. Nat. Neurosci. 11, 1109–1116. doi: 10.1038/nn.2182, 19160510

[ref2] Chen Y. Zulnaidi H. Syed Ali S. K. B. (2023). Study on the eye movement characteristics of the badminton practitioners of different levels regarding visual attention. Front. Psychol. 13:1026006. doi: 10.3389/fpsyg.2022.1026006, 36875544 PMC9983635

[ref3] Costa S. Berchicci M. Bianco V. Croce P. Di Russo F. Quinzi F. . (2023). Brain dynamics of visual anticipation during spatial occlusion tasks in expert tennis players. Psychol. Sport Exerc. 65:102335. doi: 10.1016/j.psychsport.2022.102335, 37665843

[ref4] De Waelle S. Robertson-Martens K. Deconinck F. J. A. Lenoir M. (2023). The use of contextual information for anticipation of badminton shots in different expertise levels. Res. Q. Exerc. Sport 94, 15–23. doi: 10.1080/02701367.2021.1934378 35040748

[ref5] DeCouto B. S. Bilalić M. Williams A. M. (2024). Neuroimaging and perceptual-cognitive expertise in sport: a narrative review of research and future directions. Neuropsychologia 205:109032. doi: 10.1016/j.neuropsychologia.2024.109032, 39505198

[ref6] Dendauw E. Evans N. J. Logan G. D. Haffen E. Bennabi D. Gajdos T. . (2024). The gated cascade diffusion model: an integrated theory of decision making, motor preparation, and motor execution. Psychol. Rev. 131, 825–857. doi: 10.1037/rev0000464, 38386394 PMC7616365

[ref7] Gold J. I. Shadlen M. N. (2007). The neural basis of decision making. Annu. Rev. Neurosci. 30, 535–574. doi: 10.1146/annurev.neuro.29.051605.113038, 17600525

[ref8] Gredin N. V. Broadbent D. P. Thomas J. L. Williams A. M. (2023). The role of action tendencies in expert anticipation. Asian J. Sport Exer. Psychol. 3, 30–38. doi: 10.1016/j.ajsep.2023.02.001

[ref9] Hanks T. D. Ditterich J. Shadlen M. N. (2006). Microstimulation of macaque area LIP affects decision-making in a motion discrimination task. Nat. Neurosci. 9, 682–689. doi: 10.1038/nn1683, 16604069 PMC2770004

[ref10] Harris D. J. Arthur T. Broadbent D. P. Wilson M. R. Vine S. J. Runswick O. R. (2022). An active inference account of skilled anticipation in sport: using computational models to formalise theory and generate new hypotheses. Sports Med. 52, 2023–2038. doi: 10.1007/s40279-022-01689-w, 35503403 PMC9388417

[ref11] Harris D. J. North J. S. Runswick O. R. (2023). A bayesian computational model to investigate expert anticipation of a seemingly unpredictable ball bounce. Psychol. Res. 87, 553–567. doi: 10.1007/s00426-022-01687-7, 35610392 PMC9929032

[ref12] He H. Wang J. Ren P. Miao H. Chi L. (2025). Decoding deception: the impact of expertise and prior information on sports anticipation through computational modelling. Psychol. Sport Exerc. 78:102819. doi: 10.1016/j.psychsport.2025.102819, 39904406

[ref13] Henrich F. Hartmann R. Pratz V. Voss A. Klauer K. C. (2024). The seven-parameter diffusion model: an implementation in Stan for bayesian analyses. Behav. Res. Methods 56, 3102–3116. doi: 10.3758/s13428-023-02179-1, 37640960 PMC11133036

[ref14] Hu Z. Zhang Y. Dong T. Dong M. Kim S. Kim Y. (2023). Gender differences in neuromuscular control during the preparation phase of single-leg landing task in badminton. J. Clin. Med. 12:3296. doi: 10.3390/jcm12093296, 37176736 PMC10179252

[ref15] Huesmann K. H. K. Loffing F. (2024). Perception-action coupling in anticipation research: a classification and its application to racket sports. Front. Psychol. 15:1396873. doi: 10.3389/fpsyg.2024.1396873, 39108427 PMC11300321

[ref16] Johnson D. J. Hopwood C. J. Cesario J. Pleskac T. J. (2017). Advancing research on cognitive processes in social and personality psychology: a hierarchical drift diffusion model primer. Soc. Psychol. Personal. Sci. 8, 413–423. doi: 10.1177/1948550617703174

[ref17] Khalaji M. Aghdaei M. Farsi A. Piras A. (2022). The effect of eye movement sonification on visual search patterns and anticipation in novices. J. Multimodal User Interfaces 16, 173–182. doi: 10.1007/s12193-021-00381-z

[ref18] Kredel R. Hernandez J. Hossner E.-J. Zahno S. (2023). Eye-tracking technology and the dynamics of natural gaze behavior in sports: an update 2016–2022. Front. Psychol. 14:1130051. doi: 10.3389/fpsyg.2023.1130051, 37359890 PMC10286576

[ref19] Liu Y. Qin Y. Zhang Q. Liu H. Li J. Zhang Y. (2024). Cue depth influences badminton players’ inhibition of return in 3-D static and dynamic scenarios. Acta Psychol. 248:104368. doi: 10.1016/j.actpsy.2024.104368, 38936232

[ref20] Lu Y. Huang Y. Wang D. Li D. Luan M. (2025). Preserved use of prior information under time constraints: an EEG study of action anticipation in expert athletes. Cogn. Res. Princ. Implic. 10:74. doi: 10.1186/s41235-025-00685-8, 41139757 PMC12554856

[ref21] Magnaguagno L. Zahno S. Kredel R. Hossner E.-J. (2022). Contextual information in situations of uncertainty: the value of explicit-information provision depends on expertise level, knowledge acquisition and prior-action congruency. Psychol. Sport Exerc. 59:102109. doi: 10.1016/j.psychsport.2021.102109

[ref22] Mou H. Liu L. Wang Y. (2025). Motor expertise shapes crossmodal and modality-specific action representations in table tennis players. Behav. Brain Funct. 21:34. doi: 10.1186/s12993-025-00296-9, 41034983 PMC12487376

[ref23] Müller S. Morris-Binelli K. Hambrick D. Z. Macnamara B. N. (2024). Accelerating visual anticipation in sport through temporal occlusion training: a meta-analysis. Sports Med. 54, 2597–2606. doi: 10.1007/s40279-024-02073-6, 39102157 PMC11467115

[ref24] Myers C. E. Interian A. Moustafa A. A. (2022). A practical introduction to using the drift diffusion model of decision-making in cognitive psychology, neuroscience, and health sciences. Front. Psychol. 13:1039172. doi: 10.3389/fpsyg.2022.1039172, 36571016 PMC9784241

[ref25] Pan W. Geng H. Zhang L. Fengler A. Frank M. J. Zhang R.-Y. . (2025). dockerHDDM: a user-friendly environment for Bayesian hierarchical drift-diffusion modeling. Adv. Methods Pract. Psychol. Sci. 8:25152459241298700. doi: 10.1177/25152459241298700

[ref26] Paolini S. Bazzini M. C. Rossini M. De Marco D. Nuara A. Presti P. . (2023). Kicking in or kicking out? The role of the individual motor expertise in predicting the outcome of rugby actions. Front. Psychol. 14:1122236. doi: 10.3389/fpsyg.2023.1122236, 36935992 PMC10020490

[ref27] Paolini S. Presti P. Scalona E. Boccolini G. Rizzolatti G. Fabbri-Destro M. . (2025). The individual training history shapes soccer players’ ability to predict teammates’ and opponents’ moves. Sci. Rep. 15:13057. doi: 10.1038/s41598-025-85130-y, 40240350 PMC12003635

[ref28] Rizzolatti G. Craighero L. (2004). The mirror-neuron system. Annu. Rev. Neurosci. 27, 169–192. doi: 10.1146/annurev.neuro.27.070203.144230, 15217330

[ref29] Robertson-Martens K. De Waelle S. Deconinck F. Lenoir M. (2022). Differences in expertise level for anticipatory skill between badminton “in game” strokes and serves. Int. J. Sports Sci. Coaching 17, 782–791. doi: 10.1177/17479541211046910

[ref30] Servant M. Logan G. D. Gajdos T. Evans N. J. (2021). An integrated theory of deciding and acting. J. Exp. Psychol. Gen. 150, 2435–2454. doi: 10.1037/xge0001063, 34370503

[ref32] Smith D. M. (2016). Neurophysiology of action anticipation in athletes: a systematic review. Neurosci. Biobehav. Rev. 60, 115–120. doi: 10.1016/j.neubiorev.2015.11.007, 26616736

[ref33] Song T. Ye M. Teng G. Zhang W. Chen A. (2025). The role of action anticipation in specific sport performance: a three-level meta-analysis and systematic review in temporal occlusion paradigm. Psychol. Sport Exerc. 79:102839. doi: 10.1016/j.psychsport.2025.102839, 40090558

[ref34] Wang X. Ren P. Miao X. Chi L. (2025). Multisensory training enhances anticipation skills in badminton novices. Sci. Rep. 15:9862. doi: 10.1038/s41598-025-93475-7, 40119081 PMC11928664

[ref35] Weinberg H. Müller F. Cañal-Bruland R. (2025). Context modulates evidence accumulation in split-second handball penalty decisions. Cogn. Res. 10:2. doi: 10.1186/s41235-025-00615-8, 39900736 PMC11790534

[ref36] Wiecki T. V. Sofer I. Frank M. J. (2013). HDDM: hierarchical Bayesian estimation of the drift-diffusion model in Python. Front. Neuroinformat. 7:14. doi: 10.3389/fninf.2013.00014, 23935581 PMC3731670

[ref37] Yan Z. Wang J. Chen Y. Lu Y. (2025). Motor experience enhances dimension-specific action anticipation: evidence from electroencephalography and computational modeling. Psychol. Sport Exerc. 82:102999. doi: 10.1016/j.psychsport.2025.102999, 40992678

[ref38] Zhao J. Gu Q. Zhao S. Mao J. (2022). Effects of video-based training on anticipation and decision-making in football players: a systematic review. Front. Hum. Neurosci. 16:945067. doi: 10.3389/fnhum.2022.945067, 36438631 PMC9686440

